# Massive Ascites in a Renal Transplant Patient after Laparoscopic Fenestration of a Lymphocele

**DOI:** 10.1155/2016/7491627

**Published:** 2016-11-07

**Authors:** Shohei Kawaguchi, Takahiro Nohara, Takashi Shima, Satoko Matsuyama, Chikako Nose, Junya Yamahana, Yoshifumi Kadono, Chikashi Seto, Masahiko Kawabata, Atsushi Mizokami

**Affiliations:** ^1^Department of Urology, Toyama Prefectural Central Hospital, Toyama, Japan; ^2^Department of Integrative Cancer Therapy and Urology, Kanazawa University Graduate School of Medical Science, Kanazawa, Japan; ^3^Department of Internal Medicine, Toyama Prefectural Central Hospital, Toyama, Japan

## Abstract

Retroperitoneal lymphocele is a common complication of renal transplantation. Here, we report the case of a 67-year-old woman with massive ascites after fenestration surgery for a lymphocele that developed following renal transplantation. She had been on continuous ambulatory peritoneal dialysis for 9 years. Living donor renal transplantation was performed and an intrapelvic lymphocele subsequently developed. The lymphocele did not resolve after aspiration therapy; therefore, laparoscopic fenestration was performed. Although the lymphocele disappeared, massive ascites appeared in its stead. Half a year later, the ascites was surgically punctured, which then gradually resolved and disappeared 6 weeks later. Aspiration therapy should be considered in patients on long-term peritoneal dialysis, although laparoscopic fenestration is safe and effective.

## 1. Introduction

Retroperitoneal lymphocele is a common complication after renal transplantation. The incidence rate of lymphocele after renal transplantation is 1%–26% [1]. The lymphocele is usually small, asymptomatic, and incidentally diagnosed during routine ultrasonography. However, symptomatic lymphoceles may affect graft function and thus require treatment. The treatment options for lymphoceles occurring after renal transplantation include simple aspiration, sclerotherapy, and drain placement. Laparoscopic and open peritoneal fenestration surgeries are more invasive options. Previous studies found that laparoscopic fenestration is a safe, effective, and possible first-line treatment for larger, symptomatic lymphoceles when there is a high risk for graft dysfunction [2, 3].

To the best of our knowledge, this is the first report of massive ascites developing in a renal transplant patient after peritoneal fenestration surgery for a symptomatic lymphocele.

## 2. Case Presentation

A 67-year-old woman who had been on continuous ambulatory peritoneal dialysis (CAPD) for 9 years received a living donor renal transplant. There was no history of peritonitis during CAPD. Graft function was good and her serum creatinine level was 0.9 mg/dL 5 days after the transplantation. The patient received combination immunosuppressive therapy with methylprednisolone, tacrolimus, mycophenolate mofetil, and basiliximab.

**Figure 1:**
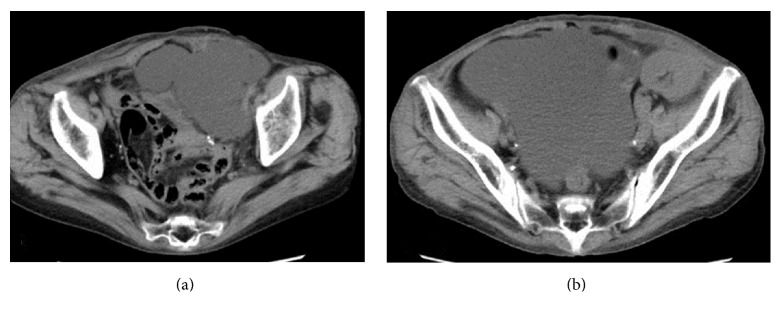
The lymphocele developed from around the graft to the front of the bladder after transplantation (a). The ascites developed and increased after fenestration surgery (b).

Perirenal fluid collection, detected 8 days after transplantation during routine ultrasonography, increased on a daily basis. On day 14, simple aspiration was performed as lower abdominal pressure was increasing. The creatinine level of the drainage was equivalent to that of the serum. After aspiration, fluid immediately reaccumulated in the perirenal space (Figure 1(a)). On day 32, laparoscopic surgery to fenestrate the lymphocele into the peritoneal cavity was performed by introducing a ~5 cm incision in the peritoneum. The septa of the lymphocele were then removed.

Three days after fenestration, the lymphocele had almost completely resolved. However, ascites was detected with ultrasonography 5 days after fenestration, which gradually increased. On day 79, abdominal computed tomography was performed because of intense abdominal fullness (Figure 1(b)). Massive ascites was detected and a diagnostic puncture was performed. Laboratory analysis of the fluid revealed the following characteristics: specific gravity (1.017), total protein concentration (2.8 g/dL), Rivalta reaction (negative), and ratio of ascites to serum lactate dehydrogenase (0.5). Culture and cytodiagnosis of the ascites and a T-Spot tuberculosis assay were all negative. The patient was followed up as an outpatient because the ascites was transudative and not cancerous or tuberculous.

**Figure 2:**
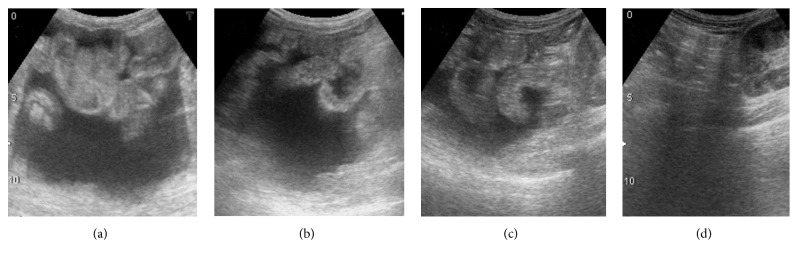
Lower abdominal ultrasonography. (a) Before percutaneous abdominal puncture; (b) 2 weeks after abdominal puncture; (c) 4 weeks after abdominal puncture; and (d) 6 weeks after abdominal puncture (ascites completely resolved).

On day 163 after fenestration, the patient presented to the outpatient clinic with prominent abdominal fullness. A percutaneous abdominal puncture was performed and 2 L of ascitic fluid was aspirated. Two weeks after aspiration, the ascites significantly decreased and the abdominal fullness was resolving. The ascites continued to decrease and disappeared 6 weeks after aspiration (Figure 2). There was no recurrence after 2.5 years of follow-up.

## 3. Discussion

Lymphocele after renal transplantation results from transection of the lymphatic vessels accompanying the external iliac vessels during transplantation surgery and subsequent lymph accumulation in a nonepithelized retroperitoneal cavity. Dissection around the external iliac vessels is necessary to perform vascular anastomoses. However, extensive dissection is a risk factor for lymphocele formation [4]. Limited dissection and careful ligation of iliac lymphatic vessels decrease the incidence rate of lymphoceles [5].

Management of lymphocele after renal transplantation is variable. Aspiration therapy is performed to reduce leakage of lymphatic fluid, and fenestration surgery is performed to allow the peritoneum to absorb the fluid. Laparoscopic surgery to fenestrate the lymphocele into the peritoneal cavity is preferred because the rate of recurrence (8%) is lower than that with open surgery (16%) and aspiration therapy (59%) [1].

Long-term CAPD can lead to structural changes in the peritoneal membrane, such as loss of mesothelial cells, thickening of the submesothelial zone, and alterations of blood vessel walls [6]. Functional transport abnormalities of the peritoneal membrane, such as loss of ultrafiltration, occur subsequent to these structural changes [7].

In the present case, we performed laparoscopic fenestration after simple aspiration. Although the lymphocele disappeared, a large quantity of ascites developed. Nine years of CAPD may have produced structural changes in and loss of function of the peritoneal membrane. A massive amount of lymph fluid then leaked from around the external iliac vessels and accumulated in the peritoneal cavity without absorption. A percutaneous abdominal puncture was performed 5 months after fenestration, and the ascites subsequently decreased and disappeared 6 weeks after aspiration. The resolution of the ascites may have been caused by the decrease in lymph fluid leakage due to long-term high abdominal pressure.

Laparoscopic fenestration is a safe and effective first-line treatment for symptomatic lymphocele occurring after renal transplantation. However, aspiration therapy, such as percutaneous catheter drainage with or without sclerotherapy, should be considered for patients with peritoneal function that is impaired by long-term peritoneal dialysis.
